# Radiomics and the Image Biomarker Standardisation Initiative (IBSI): A Narrative Review Using a Six-Question Map and Implementation Framework for Reproducible Imaging Biomarkers

**DOI:** 10.7759/cureus.95335

**Published:** 2025-10-24

**Authors:** Heriberto Aguirre-Meneses, Pablo Stoehr-Muñoz, Mauricio Molina-Gonzalez, Marco-Antonio Nuñez-Gaona, Ernesto Roldan-Valadez

**Affiliations:** 1 Department of Medical Systems, Instituto Nacional de Rehabilitación "Luis Guillermo Ibarra Ibarra", Mexico City, MEX; 2 Department of Biomedical Engineering, Universidad Iberoamericana, Mexico City, MEX; 3 Division of Research, Instituto Nacional de Rehabilitación "Luis Guillermo Ibarra Ibarra", Mexico City, MEX; 4 Department of Radiology, I.M. Sechenov First Moscow State Medical University (Sechenov University), Moscow, RUS

**Keywords:** artificial intelligence in radiology, benchmarking, clinical translation, image biomarker standardisation initiative, multicenter validation, preprocessing standardization, quantitative imaging biomarkers, radiomics, reproducibility in research, texture analysis

## Abstract

Radiomics can quantify image-derived tumor heterogeneity and support diagnosis, prognosis, and treatment assessment, yet adoption has been limited by poor reproducibility across scanners, protocols, and software. The Image Biomarker Standardisation Initiative (IBSI) was founded to harmonize feature definitions and preprocessing. This narrative review explains what IBSI standardizes, how it is implemented, and what clinicians need to know, using a six-question map (who, why, what, how, when, where) and an implementation framework linking standardized pipeline blocks to compliance tools.

Targeted searches (PubMed/Google Scholar, 2010-2025) emphasized IBSI manuals, consensus statements, multicenter evaluations, and clinically oriented studies. Because this is a narrative review, no quantitative synthesis (meta-analysis or meta-regression) was performed; numerical examples (e.g., an entropy coefficient-of-variation illustration) are descriptive.

IBSI specifies the mathematics of radiomic features and upstream steps (interpolation/re-segmentation, intensity discretization, convolutional filtering, and feature aggregation) and provides compliance resources (digital phantoms, benchmark values, validation checklists/portals). These measures improve cross-software agreement - especially for first-order and shape features - while clarifying residual variability in higher-order textures. IBSI complements QIBA (acquisition/reconstruction profiles) and DICOM (data/metadata standards) and is now embedded in widely used platforms (e.g., PyRadiomics, LIFEx), enabling more stable multicenter workflows and integration with machine-learning models.

IBSI functions as a clinical quality infrastructure for radiomics. Adopting its standardized pipeline and compliance framework reduces inter-software variability, strengthens the generalizability of predictive and prognostic models, and supports regulatory readiness. Continued refinement of filters and texture metrics, together with transparent reporting and shared datasets, will further enhance reproducibility and clinical trust.

## Introduction and background

Radiomics has rapidly emerged as a quantitative imaging approach in which high-throughput features are extracted from standard CT, MRI, and PET to characterize tumor heterogeneity beyond visual assessment and to support diagnosis, prognostication, and treatment monitoring [1,2]. For clarity, we use the gray-level co-occurrence matrix (GLCM) to denote a texture matrix that quantifies the co-occurrence of intensity pairs at defined spatial offsets, and we use discretization to mean mapping continuous voxel intensities into fixed bins - either a fixed bin number or a fixed bin size - prior to texture computation.

Despite its promise, clinical adoption has been limited by reproducibility gaps: feature values can shift with acquisition and reconstruction settings, segmentation choices, and software implementations, even when the same datasets are analyzed [3-7]. These discrepancies erode confidence, complicate regulatory evaluation, and hinder multi-center studies.

Established in 2016, the Image Biomarker Standardisation Initiative (IBSI) addresses these limitations by formalizing feature definitions and harmonizing preprocessing workflows. IBSI provides mathematically precise specifications, digital phantoms with benchmark values, and compliance procedures for software calibration, forming a methodological backbone that connects computational rigor to clinical needs [8,9].

IBSI complements other efforts. The Quantitative Imaging Biomarker Alliance (QIBA) focuses on modality-specific acquisition/reconstruction profiles and test-retest performance [10], whereas the DICOM standard primarily governs data exchange and metadata (e.g., segmentation and parametric map objects). In contrast, IBSI specifies the mathematics of radiomic features and the upstream preprocessing required to obtain comparable values across software [11].

IBSI’s influence extends beyond definitions: integration into widely used open-source platforms (e.g., PyRadiomics [12] and LIFEx [13]) has improved cross-platform agreement, particularly for first-order and shape features, while ongoing benchmarks highlight residual variability in higher-order texture metrics that motivates continued refinement of filters and pipelines [4,5,7,14,15]. This review synthesizes IBSI’s contributions and clinical implications, structured around who leads IBSI, why it was created, what it standardizes, how standardization is applied, when it evolved, and where it is being implemented to enhance reproducibility and clinical trust. Although prior reviews have summarized IBSI’s technical content, an integrated six-question framework tailored to clinicians and developers has been lacking; this review addresses that gap.

This narrative review is based on targeted searches (PubMed and Google Scholar, 2010-2025) using terms related to "radiomics," "IBSI," and "standardization," prioritizing consensus statements, IBSI manuals, multi-center evaluations, and clinically oriented studies; PRISMA was not applied.

No quantitative synthesis (meta-analysis or meta-regression) was performed; therefore, pooled effect estimates, P-values, and confidence intervals are not reported. Numerical examples in some figures are illustrative and descriptive, not inferential.

## Review

Radiomics in clinical imaging: What it is and why it matters

Radiomics refers to the high-throughput extraction of quantitative features from routine CT, MRI, and PET examinations to characterize disease phenotypes beyond visual assessment. Feature families include first-order intensity statistics, geometric shape descriptors, multiple texture matrices, and filter-derived measures intended to capture tumor heterogeneity in a reproducible, quantitative manner [1,2,16].

Over the past decade, radiomics has become integral to precision medicine. When combined with robust statistical modeling and machine-learning pipelines, radiomic signatures can support patient stratification, predict treatment response, and, in selected contexts, infer genomic or histopathologic profiles non-invasively [6,17].

Despite promising results, routine clinical adoption remains limited. Feature values can vary substantially with acquisition and reconstruction parameters, segmentation strategies, and software implementations - even when identical datasets are analyzed - undermining generalizability and complicating regulatory evaluation [3,18].

Radiomics should not be conflated with artificial intelligence. Classical radiomics relies on handcrafted, predefined features, whereas deep learning derives representations automatically from data. Hybrid approaches increasingly integrate radiomic descriptors with AI to balance interpretability and performance [19].

These methodological inconsistencies motivated the creation of the Image Biomarker Standardisation Initiative, a collaborative effort that harmonizes feature definitions and preprocessing workflows to improve reproducibility and clinical credibility.

Origins and governance of IBSI: Why it was created and who leads it

The IBSI was founded in 2016 to address pervasive reproducibility failures that hampered early radiomics research. Identical datasets frequently yielded discordant feature values due to unstandardized feature definitions, heterogeneous preprocessing, and undocumented software behavior [20]. Additional problems, such as vague or conflicting nomenclature, limited transparency of software pipelines, and the absence of reference values for verification, further undermined external validation and clinical confidence [21]. To resolve these issues, IBSI defined mathematically rigorous feature specifications and standardized terminology, provided reference datasets and digital phantoms, and laid out a reproducible image-processing framework spanning acquisition, interpolation, discretization, and feature calculation [14,22]. IBSI also instituted compliance benchmarking: participating groups computed features with their own software and compared results with IBSI reference values, exposing inter-platform discrepancies and enabling iterative correction. These foundations created a platform for multicenter trials and for regulatory evaluation of radiomics pipelines [23] and, more broadly, helped restore scientific credibility by enabling clinically actionable, reproducible biomarkers that support personalized medicine strategies [24].

IBSI operates as a decentralized, multidisciplinary consortium of experts in medical imaging, radiomics, physics, oncology, and computational science. From its inception, leadership has been anchored by institutions such as the University Medical Center Groningen (UMCG) and the French National Institute of Health and Medical Research (INSERM), which provided early conceptual and methodological guidance [8,14]. Coordination occurs through structured working groups that comprise more than 25 research teams across Europe, North America, and Asia; these groups collaborate on phantom design, feature definition, benchmarking pipelines, and statistical validation of feature reproducibility [9,19]. Participation requires teams to implement feature-extraction pipelines independently and validate their outputs against IBSI test cases, using iterative feedback and open benchmarking to refine definitions and ensure cross-platform agreement [9,19]. Key contributors include Andriy Zwanenburg, whose leadership has been central to the reference documentation and methodology, and Matthieu Hatt, Ivana Buvat, Esther Troost, and Hugo J.W.L. Aerts, internationally recognized for work in radiomics and translational imaging [25]. IBSI’s consensus-driven, open-science model has catalyzed broad engagement and accelerated adoption across academic and clinical software platforms.

What IBSI standardizes

The IBSI provides a comprehensive framework for radiomics standardization that spans mathematically precise feature definitions and the upstream image-processing workflow required for their computation. Its overarching goal is to reduce inter-software and inter-institutional variability and thereby improve reproducibility across platforms and protocols [26].

IBSI has published standardized definitions for more than 150 radiomic features, organized into major families: shape (morphology), first-order statistics, intensity-volume histogram, and texture matrices such as the gray-level co-occurrence matrix (GLCM), gray-level run length matrix (GLRLM), and gray-level size zone matrix (GLSZM). Each feature is accompanied by a formal mathematical formulation, aggregation strategy, and expected behavior under defined conditions [27]. For an overview of feature classes, indicative compliance status, and typical clinical relevance, see Table 1.

**Table 1:** Radiomic Feature Classes Standardized by the Image Biomarker Standardisation Initiative (IBSI). This table summarizes the primary radiomic feature classes standardized by IBSI, including the approximate number of features per class, their compliance status, representative examples, and typical clinical relevance. Shape descriptors, first-order statistics, and texture matrices (e.g., GLCM, GLRLM, GLSZM, NGTDM, GLDM) have been fully standardized with published mathematical definitions, phantom validation, and benchmark values. Filtered and higher-order/custom features remain partially or not standardized and therefore require careful implementation and interpretation across radiomics platforms. Compliance status definitions: Fully standardized—mathematical definitions, benchmark values, and phantom datasets published by IBSI; Partially standardized—feature class recognized, but implementation details may vary across software tools; Not standardized—no official IBSI guidelines or benchmark datasets currently available. Table created by the authors, summarized data from the IBSI 1 [14]. Abbreviations: GLCM, gray-level co-occurrence matrix; GLRLM, gray-level run length matrix; GLSZM, gray-level size zone matrix; NGTDM, neighborhood gray-tone difference matrix; GLDM, gray-level dependence matrix; LoG, Laplacian of Gaussian

Feature Class	Approx. No. of Features	IBSI Compliance Status	Representative Features	Typical Clinical Relevance
Shape-based	16	Fully standardized	Volume, surface area, compactness, sphericity	Tumor burden; surgical planning
First-order (Intensity)	19	Fully standardized	Mean, median, entropy, skewness, kurtosis	Tumor heterogeneity; therapy response
GLCM	24	Fully standardized	Contrast, correlation, homogeneity, energy	Texture pattern analysis (e.g., fibrosis)
GLRLM	16	Fully standardized	Short run emphasis, run length non-uniformity	Tumor tissue texture evaluation
GLSZM	16	Fully standardized	Zone entropy, large zone emphasis	Necrosis; tissue density characterization
NGTDM	5	Fully standardized	Coarseness, busyness, complexity	Structural patterns; inflammation markers
GLDM	14	Fully standardized	Dependence entropy, high gray-level emphasis	Tumor consistency; infiltration signatures
Filtered (e.g., wavelets, LoG)	Varies by implementation	Partially standardized	Wavelet-energy, LoG-entropy	Multi-scale analysis; fine texture features
Higher-order or custom	Variable	Not standardized	Fractal dimension, laws’ texture energy	Experimental/AI feature engineering

Standardization also covers essential preprocessing steps: voxel interpolation, image resegmentation, intensity discretization, and the application of spatial filters. In particular, two discretization schemes, fixed bin number (FBN) and fixed bin size (FBS), are formalized because they materially influence texture metrics and must be transparently reported to ensure comparability [26,28].

The workflow is described in modular form with step-by-step guidance and is complemented by reference phantoms and datasets (digital and computational) with known ground-truth values for validation and calibration of software implementations [29]. More recently, IBSI guidance has expanded to commonly used convolutional filters, including wavelet, Laplacian of Gaussian (LoG), and Gabor, to support multi-scale and orientation-aware feature extraction with public benchmark examples where available [26].

Finally, IBSI provides practical resources for verifying software compliance. Excel-based reference sheets, online validation portals, and comparative benchmarks enable quantitative assessment; tools are considered compliant when feature values fall within narrow tolerances relative to IBSI reference values [14,30].

**Figure 1:**
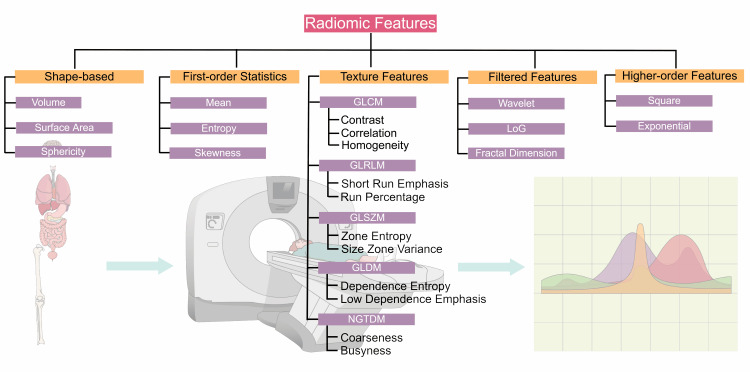
IBSI-based radiomic feature taxonomy. Hierarchical taxonomy of radiomic features according to the Image Biomarker Standardisation Initiative (IBSI). Features are grouped into five classes: shape-based, first-order statistics, texture features, filtered features, and higher-order features. Texture features are subdivided into matrix families (GLCM, GLRLM, GLSZM, GLDM, NGTDM). Example metrics (e.g., contrast, entropy, sphericity) illustrate common clinical use. The taxonomy mirrors IBSI formal definitions and benchmarked implementations. Original schematic created by the authors using the Mind the Graph platform (www.mindthegraph.com); not reproduced from previously published material; no third-party permissions required. Abbreviations: IBSI, Image Biomarker Standardisation Initiative; GLCM, gray-level co-occurrence matrix; GLRLM, gray-level run length matrix; GLSZM, gray-level size zone matrix; GLDM, gray-level dependence matrix; NGTDM, neighborhood gray-tone difference matrix

By unifying quantitative definitions with a transparent preprocessing workflow, IBSI offers an end-to-end ecosystem for reproducible feature extraction. This structure has become foundational for radiomics studies aiming for regulatory credibility and clinical impact. A hierarchical taxonomy of IBSI-standardized feature classes is illustrated in Figure 1.

How IBSI standardization is applied

The practical implementation of IBSI standardization begins with a rigorously specified image-processing pipeline designed to ensure reproducible feature extraction. IBSI emphasizes key operations - image padding, convolutional filtering, and feature aggregation - implemented under explicit computational rules and reporting conventions [26,31].

**Figure 2:**
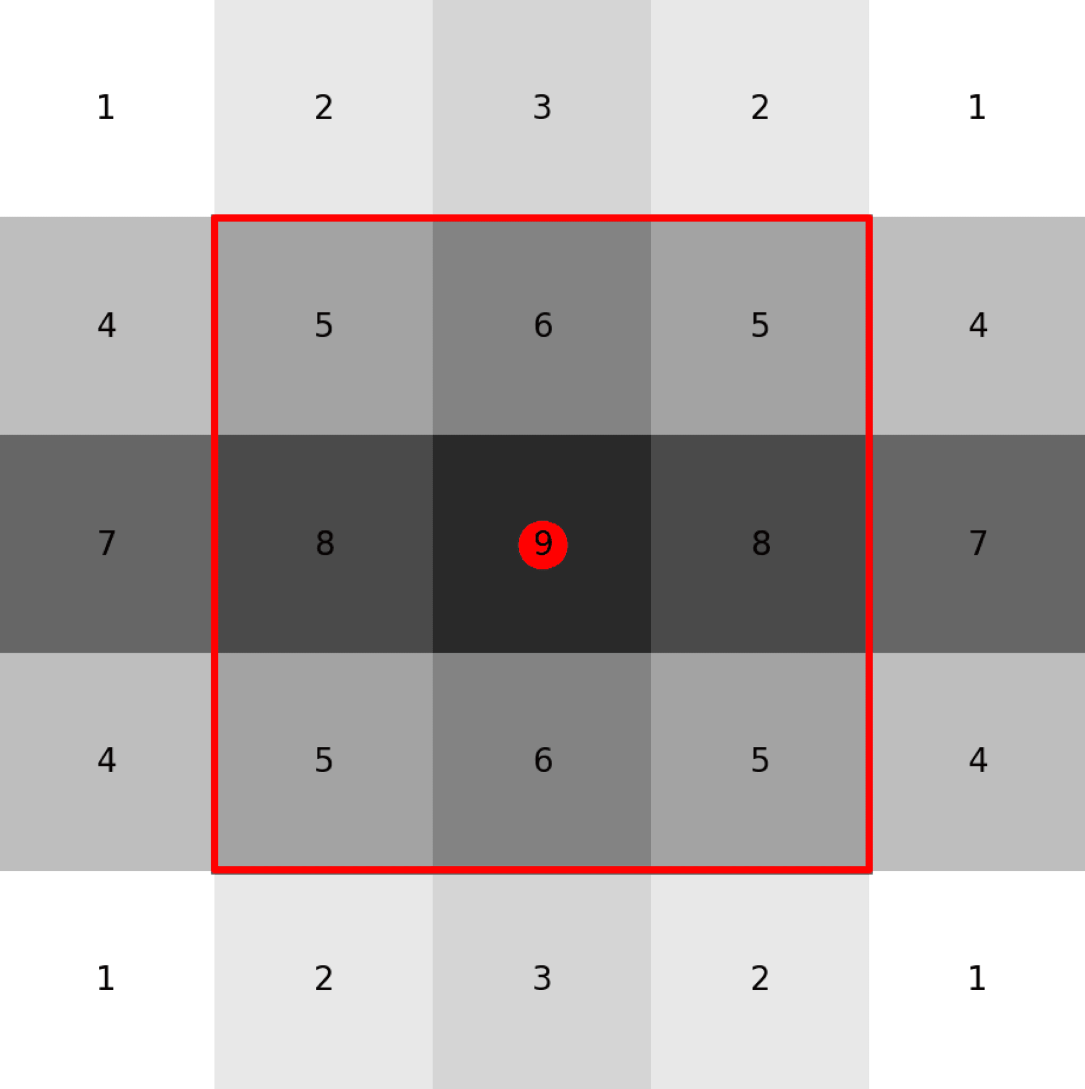
Discrete convolution on a 2D image patch. Illustration of a 5x5 intensity matrix with a 3x3 kernel centered at ko. The red circle marks the evaluation position ko; the red rectangle shows the kernel window. The output value h[ko] equals the weighted sum of the intensities within the window and the kernel coefficients, demonstrating how the convolution response is computed at a specific spatial location. Original schematic created by the authors using the Mind the Graph platform (www.mindthegraph.com); not reproduced from previously published material; no third-party permissions required.

At the core of this process is the discrete convolution, formalized as follows:

$h\left\lceil k_{0} \right\rceil=(g*f)\left\lfloor k_{0} \right\rfloor\sum_{_{k\in M^{D}}}^{}g\left\lfloor k \right\rfloor f\left\lceil k_{0}-k \right\rceil$

where f[k] represents the input image, g[k] the convolutional filter, and h[k] the resulting response map. The term k0 is the index (or coordinate) of the pixel or voxel in the image domain where the convolution is calculated, and k is the sum variable (index that runs through the filter values). Figure 2 shows a 2D image with a kernel centered at k0. This operation is applied across the entire image domain using padding strategies such as zero, mirror, or nearest-neighbor extensions to mitigate boundary artifacts [32].

That is why the IBSI devotes significant effort to standardizing convolutional filters, as they are an essential preliminary step in generating derived images that are then used to calculate radiomic features. This makes convolution a standardized block within the pipeline, ensuring that different platforms produce the same feature values from the same images.

It is true that convolution as a mathematical operation was not invented to "guarantee reproducibility"; however, the standardized definition of its implementations in IBSI (kernels, padding, normalization) is what ensures that different software and research centers produce consistent and comparable results, as demonstrated by multicenter validation studies [1].

Once filtered, the response map h[k] is not directly interpretable as a radiomic feature. It must first be aggregated over a predefined region of interest (ROI). A commonly used aggregation formula computes the mean response within the ROI:

η = (1/N) ∑ₖ∈ROI h[k]

This scalar η then serves as the input for downstream statistical or machine-learning models [32].

IBSI further prescribes specific classes of convolutional filters, including LoG, wavelets, and Gabor filters, each characterized by a defined frequency response profile. These filters enable multi-scale and multi-orientation analysis, which is essential for capturing complex tissue heterogeneity [33].

**Figure 3:**
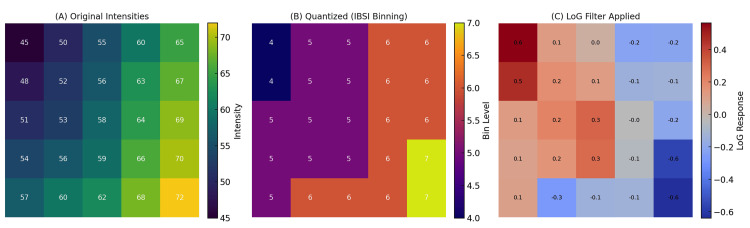
IBSI preprocessing and filtering workflow. (A) Original image patch with continuous voxel intensities. (B) IBSI-compliant gray-level discretization (fixed bin width) to standardize intensity representation. (C) LoG-filtered image emphasizing structures at a defined spatial scale. The sequence (discretization → filtering) exemplifies how IBSI standardization removes arbitrary intensity rescaling and yields analysis-ready images for reproducible feature extraction. Original schematic created by the authors using the Mind the Graph platform (www.mindthegraph.com); not reproduced from previously published material; no third-party permissions required. Abbreviations: IBSI, Image Biomarker Standardisation Initiative; LoG, Laplacian-of-Gaussian

A visual demonstration of this process is presented in Figure 3, which illustrates the sequential steps recommended by IBSI for preparing imaging data prior to radiomic feature extraction. Panel A depicts the original voxel intensity distribution; Panel B shows IBSI-compliant gray-level discretization using a fixed bin width; and Panel C presents a LoG-filtered image that accentuates structural patterns across spatial scales. This example underscores how discretization and convolutional filtering operate together to enhance reproducibility and capture clinically relevant heterogeneity.

Another pillar of IBSI standardization is rotation invariance, achieved through techniques such as steerable filtering and orientation pooling. These methods combine responses from rotated filter instances and apply voxel-wise maxima or averages to neutralize angular bias while preserving essential structural information [32].

By formalizing these preprocessing steps, IBSI provides a robust foundation for developing compliant radiomic software. This level of standardization is indispensable for achieving cross-platform feature reproducibility, enabling regulatory validation, and promoting clinical translation.

When was IBSI established, and how did it evolve?

The IBSI was formally launched in 2016 to remedy methodological inconsistency and enable reproducible radiomic feature extraction at scale. Early deliverables focused on synthetic phantoms and reference feature definitions, providing the first common ground for comparing independently developed software implementations [34].

IBSI’s development then progressed through phases: first, standardizing the mathematical definitions of features and validating them on digital phantoms; next, extending to clinical CT datasets processed with standardized pipelines to test feature robustness in practice. By the conclusion of this cycle, more than 170 features had been validated across multiple software teams, with strong inter-platform agreement for most classes [9,35].

**Figure 4:**
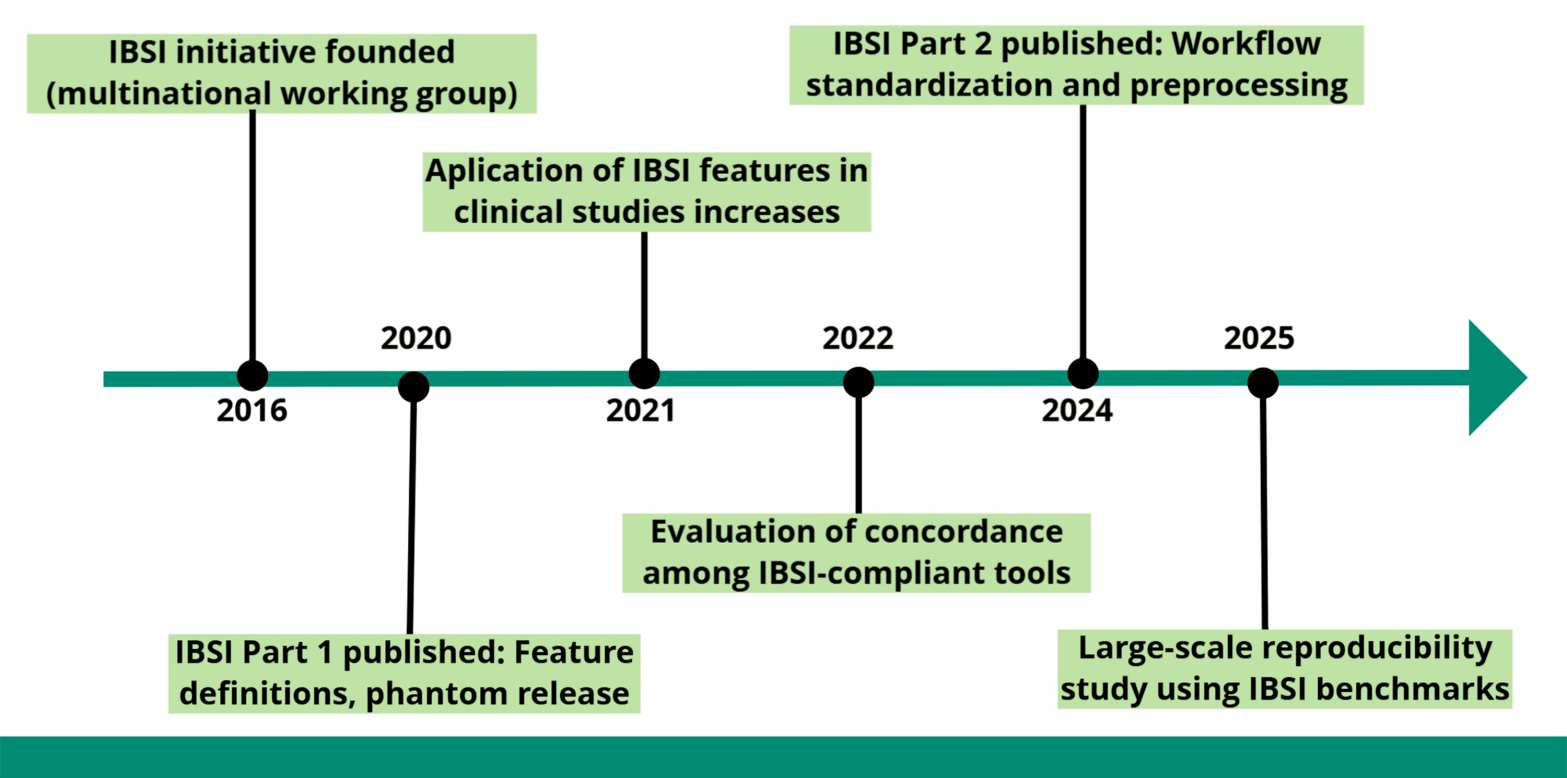
Timeline of IBSI development and milestones. Chronology of major IBSI milestones: inception (2016); standardized radiomic feature definitions (2020); early clinical implementation and cross-platform evaluations (2021–2022); publication of IBSI Part 2 with formalized preprocessing workflows (2024); and expanded multicenter reproducibility validation using benchmark phantoms and datasets (2025). The timeline highlights IBSI’s growing role in aligning radiomics with clinical and regulatory expectations. Original schematic created by the authors using the Mind the Graph platform (www.mindthegraph.com); not reproduced from previously published material; no third-party permissions required.

A major advance arrived with IBSI Part 2 (2024), which formalized the preprocessing workflow (e.g., discretization, interpolation, filtering), expanded test phantoms, and released benchmark values for CT, MRI, and PET pipelines [8]. The updated guidance emphasized transparent, step-wise reporting and promoted software compliance documentation for cross-center use [36,37]. These resources, together with the general workflow specification already adopted by many toolkits, continue to support calibration, quality assurance, and reproducibility checks across platforms [26]. Key milestones and their chronology are summarized in Figure 4.

Where is IBSI applied and recognized?

Research Implementation and Software Compliance

IBSI guidance has been incorporated into widely used radiomics platforms (open source), e.g., PyRadiomics, LIFEx, SERA (https://github.com/ashrafinia/SERA), RaCaT (https://github.com/EPfaehler/RaCat), enabling standardized feature-extraction pipelines across centers. Comparative evaluations consistently show higher agreement for shape and first-order features, with residual variability for higher-order texture metrics, underscoring the value of standardization and the need for continued refinement [36].

Clinical Application Across Modalities

IBSI-compliant workflows have been applied in clinical research using CT, MRI, and PET. Pilot multi-institution projects in head and neck cancer and soft-tissue sarcoma reported improved feature stability across institutions and acquisition protocols [38,39]. In diffuse midline glioma with the H3F3A K27M (H3.3K27M) mutation, carefully selected MRI-derived first- and second-order radiomics distinguished viable tumor and peritumoral edema from equivalent normal tissue, illustrating the translational potential of standardized pipelines in neuro-oncology [40]. IBSI-aligned feature extraction is also being evaluated in digital pathology and photoacoustic imaging, indicating expanding applicability beyond traditional radiology [41].

Reproducibility Audits and Consensus Projects

IBSI now serves as a reference framework for reproducibility audits in radiomics and AI-based biomarker development. Standardized phantom studies and international consensus efforts have employed IBSI benchmarking tools to assess inter-platform concordance and to guide quality assurance in multicenter research, recommending IBSI adherence for clinical and regulatory readiness [42].

Global Visibility and Validation Resources

IBSI’s open-access validation portal (https://ibsi.radiomics.hevs.ch) provides benchmarking datasets, certified feature-value checklists, and compliance verification tools, widely used to document reproducibility across computational environments [30].

Together, these implementations embed IBSI principles in research workflows, clinical feasibility studies, and reproducibility assessments, enabling consistent biomarker extraction for AI applications and evidence-based clinical decision-making. Representative clinical studies using IBSI-compliant workflows are summarized in Table 2.

**Table 2:** Clinical studies and use-cases employing IBSI-compliant radiomics pipelines. This table summarizes representative clinical studies and real-world use-cases that have implemented radiomics workflows aligned with the IBSI. Each entry specifies the target organ or pathology, imaging modality, radiomic feature classes analyzed, the associated clinical objective, and the method of IBSI compliance. Compliance methods include the use of certified IBSI-compliant software tools (e.g., PyRadiomics, LIFEx), benchmarking with digital phantoms, or manual adherence to standardized feature definitions. Clinical applications span diagnosis, outcome prediction, treatment response assessment, and reproducibility audits. Modalities include CT, MRI, and PET imaging. Table created by the authors; references for the studies are listed in the "Organ/Site" column. Abbreviations: GLCM, gray-level co-occurrence matrix; GLRLM, gray-level run length matrix; GLSZM, gray-level size zone matrix; GLDM, gray-level dependence matrix; NGTDM, neighborhood gray-tone difference matrix

Organ / Site	Modality	Radiomic Feature Classes Used	Clinical Objective	IBSI Compliance Method
Lung cancer (NSCLC), pulmonary embolism [43]	CT	First-order, shape, GLCM	Survival prediction	PyRadiomics (IBSI-compliant), phantom validated
Head, neck, breast cancer [43,44]	PET	GLRLM, GLSZM, NGTDM	Radiotherapy outcome assessment	In-house software aligned with IBSI definitions
Lung and liver tumors [45]	PET	First-order, GLDM, GLSZM	Feature reproducibility across scanners	LIFEx tool (IBSI-compliant), cross-platform tested
Brain metastases [46,47]	MRI	Shape, texture, filtered (wavelet)	Multi-center reproducibility audit	IBSI phantoms and benchmarking protocols
Mediastinal sarcoidosis [48,49]	PET/CT	First-order, shape	Diagnostic differentiation	Software documentation cross-verified with IBSI
Soft tissue sarcoma [50]	MRI	GLCM, GLRLM, custom	Machine learning classification	Partially IBSI-compliant tools, manual verification

How IBSI enhances reproducibility and clinical translation

Reproducibility has long limited the clinical translation of radiomics: variation in acquisition, segmentation, and feature computation has historically yielded inconsistent results, even on identical datasets. The IBSI addresses these limitations by defining radiomic features mathematically and by providing validation resources, digital phantoms, and benchmark feature values, together with transparent reporting guidance for preprocessing and aggregation [22,25,45].

Comparative evaluations indicate that IBSI-compliant pipelines achieve substantially improved agreement for first-order and shape features, categories central to clinical workflows, while highlighting areas where higher-order texture metrics still require refinement [5]. In clinical studies, these gains translate into more reliable predictive and prognostic modeling, with better generalizability across imaging centers and reduced overfitting risk when standardized workflows are used [39,40]. As a representative example, MRI-based radiomics in diffuse midline glioma with the H3F3A K27M (H3.3K27M) mutation showed strong diagnostic performance for stratifying progression-free and overall survival, with several features achieving high specificity, illustrating how IBSI-aligned pipelines can yield clinically meaningful biomarkers for aggressive CNS tumors [51].

**Figure 5:**
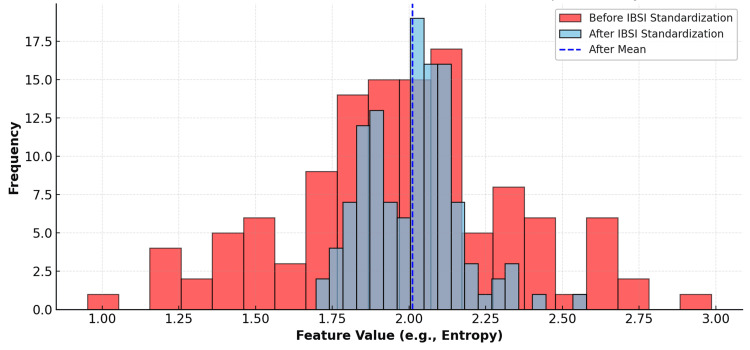
Impact of IBSI standardization on feature reproducibility. Histogram of a representative texture feature (entropy) computed across multiple software implementations before (red) and after (blue) applying Image Biomarker Standardisation Initiative (IBSI)-compliant preprocessing and feature definitions. Prior to standardization, the distribution was broad, consistent with high inter-software variability; after standardization, values converged around the mean (blue dashed line). In this illustrative sample, the coefficient of variation for entropy decreased from 34% to 7% following standardization, indicating tighter cross-software agreement. Original schematic created by the authors using the Mind the Graph platform (www.mindthegraph.com); not reproduced from previously published material; no third-party permissions required.

Beyond feature computation, IBSI influences the broader reproducibility movement in AI and quantitative imaging by encouraging comprehensive documentation of discretization, filtering, ROI handling, and feature selection, practices that align with established machine-learning and clinical-trial reporting norms [26]. IBSI’s open-access validation resources and checklists are routinely used in reproducibility audits and for software commissioning, further bridging academic development and clinical implementation [30,45]. In our illustrative dataset, the coefficient of variation for entropy decreased from 34% before standardization to 7% after applying IBSI-compliant preprocessing and feature definitions (Figure 5), illustrating the tightening of feature distributions across software. This quantitative tightening mirrors the visual convergence of the post-standardization histogram.

What clinicians and researchers need to know about IBSI

As radiomics advances toward clinical translation, clinicians, radiologists, and researchers should be familiar with the IBSI because it underpins reproducibility, transparency, and standardized reporting in imaging biomarkers. Beyond research, IBSI functions as a quality-assurance framework: by formalizing feature definitions and preprocessing workflows, it directly influences the reliability of image-derived metrics used for tumor characterization, treatment response evaluation, and prognostication, and informs trust in AI-enabled imaging tools [26,36].

Importantly, "IBSI-compliant" software may still yield different outputs if preprocessing choices (e.g., voxel resampling, interpolation, and discretization) are implemented inconsistently. Comparative evaluations show high concordance for shape and first-order features, while texture metrics can diverge across tools, highlighting the need for local commissioning and validation before clinical use [5,42].

Clinicians play a central role in enabling compliance by ensuring that acquisition and segmentation protocols are standardized and documented. IBSI’s reference phantoms, benchmark feature values, and validation checklists facilitate cross-platform audits and reproducibility assessments in practice [29,45]. From a broader clinical perspective, IBSI alignment supports multicenter studies and strengthens regulatory readiness, promoting confident adoption of radiomics models as adjuncts to established diagnostic pathways across oncology, cardiology, and neuroimaging [36,42].

In short, IBSI should be viewed not merely as a technical specification but as a clinical quality standard that fosters reliability and trust in imaging biomarkers.

## Conclusions

Reproducibility remains the decisive prerequisite for the clinical adoption of radiomics. IBSI should be viewed not merely as a technical specification but as a clinical quality infrastructure. The initiative efforts have reduced inter-software variability, established a common reference for radiomics pipelines, and strengthened the link between computational rigor and clinical practice. Standardization has yielded the clearest gains for first-order and shape features, two categories central to oncologic imaging and treatment planning, while highlighting the need for continued refinement of higher-order texture metrics.

Looking ahead, sustained progress will depend on peer-reviewed validation across modalities and vendors, open benchmarking, and consistent reporting that makes pipelines auditable and reproducible. By aligning radiomics with the expectations of multicenter studies and regulatory evaluation, IBSI enables trustworthy imaging biomarkers and supports the responsible integration of radiomics-often in combination with AI-into evidence-based patient care. Radiologists, researchers, and developers are encouraged to adopt IBSI-compliant practices and to contribute to their iterative improvement so that radiomics remains scientifically rigorous, clinically meaningful, and widely accessible.
